# Differences in the risk of immune-related pneumonitis between PD-1 and PD-L1 inhibitors: a meta-analysis according to the new mirror-principle and PRISMA guidelines

**DOI:** 10.1007/s00262-024-03736-z

**Published:** 2024-07-02

**Authors:** Yuan Tian, Zongxiu Yin, Chi Zhang, Zhuoqi Li, Yuanyuan Wang, Kai Zhang, Feng Chen, Qi Dang

**Affiliations:** 1 Phase I Clinical Research Center, Shandong Cancer Hospital and Institute, Shandong First Medical University, and Shandong Academy of Medical Sciences, No. 440, Jiyan Road, Huaiyin District, Jinan City, 250117 Shandong People’s Republic of China; 2 Radiotherapy Department, Shandong Second Provincial General Hospital, Shandong University, Jinan, 250299 Shandong People’s Republic of China; 3 Department of Pulmonary and Critical Care Medicine, Jinan Central Hospital Affiliated to Shandong First Medical University, Jinan, 250013 Shandong People’s Republic of China; 4 Department of Cardiology, The Second Hospital, Cheeloo College of Medicine, Shandong University, Jinan, 250033 Shandong People’s Republic of China; 5 Department of Oncology, The Second Affiliated Hospital of Shandong University of Traditional Chinese Medicine, Jinan, 250299 Shandong People’s Republic of China; 6 General Surgery Department, Wen-Shang County People’s Hospital, Wenshang, 272500 Shandong People’s Republic of China; 7 Department of Thoracic Surgery, Shandong Cancer Hospital and Institute, Shandong First Medical University and Shandong Academy of Medical Sciences, Jinan, 250117 Shandong People’s Republic of China

**Keywords:** PD-1, PD-L1, Pneumonitis, Mirror-principle, Meta-analysis

## Abstract

**Purpose:**

To compare the risk of immune-associated pneumonitis between PD-1 and PD-L1 inhibitors, the meta-analysis was designed.

**Method:**

The difference in risk of immune-associated pneumonitis between PD-1 and PD-L1 inhibitors was assessed by two different meta-analysis methods, the Mirror-pairing and the PRISMA guidelines.

**Results:**

A total of eighty-eight reports were used for meta-analysis, while thirty-two studies were used for the Mirror-pairing. Both PD-1 and PD-L1 inhibitors (used alone or combined with chemotherapy) increased the risk of developing immune-related pneumonitis (*P* < 0.00001; *P* < 0.00001). Based on indirect analyses results (subgroup analyses), the risk of PD-L1-induced pneumonitis was weaker than that of PD-1 inhibitors when the control group was chemotherapy (OR = 3.33 vs. 5.43) or placebo (OR = 2.53 vs. 3.19), while no obvious significant differences were found (*P* = 0.17; *P* = 0.53). For the Mirror-pairing-based meta-analysis, the risk of PD-1-induced pneumonitis was significantly higher than that of PD-L1 inhibitors (OR = 1.46, 95%CI [1.08, 1.98], *I*^2^ = 0%, *Z* = 2.47 (*P* = 0.01)). However, this difference was not significant, when they were combined with chemotherapy (OR = 1.05, 95%CI [0.68, 1.60], *I*^2^ = 38%, *Z* = 0.21 (*P* = 0.84)).

**Conclusion:**

Both PD-1 and PD-L1 inhibitors increased the risk of immune-related pneumonitis, while the risk of PD-1-induced pneumonitis was significantly higher than that of PD-L1 inhibitors.

**Supplementary Information:**

The online version contains supplementary material available at 10.1007/s00262-024-03736-z.

## Introduction

Many clinical trials have confirmed that programmed death-1 (PD-1) or Programmed cell death ligand 1 (PD-L1) inhibitors have excellent clinical efficacy for malignant tumors [1–87]. Due to their unique immune mechanism, many immune-related side effects have been reported as part of clinical trial results [1–87]. Of immunotoxic reactions, pneumonitis was mentioned and evaluated by clinicians for lung cancer patients [1–11, 13, 15, 16, 36–49, 67–71]. Due to the increasing diversity of drug combinations based on PD-1 or PD-L1 inhibitors, assessing the risk factors for pneumonitis have become much more difficult. There were no clinical trials involving a direct comparison between PD-1 and PD-L1 [1–87], which further increased the difficulty of directly comparing the differences in toxicity reactions between PD-1 and PD-L1. However, the report of the Mirror-pairing meta-analysis has made it possible for us to solve this dilemma [88, 89].

The Preferred Reporting Items for Systematic Reviews and Meta-analyses (PRISMA) reporting guideline would also be followed [90]. The results of subgroup analyses were used to test whether the analysis results of the Mirror-pairing were consistent with the PRISMA meta-analysis results. In this study, the differences in the incidence risk of pneumonitis between PD-1 and PD-L1 inhibitors were evaluated by the above two analysis methods (PRISMA and Mirror-pairing). Furthermore, the applicability and reliability of the Mirror-pairing analysis method were further validated [88–90].

## Method

The classic PRISMA analysis method was followed and prioritized for the subsequent analyses [90].

### Search strategy and screening

The searching process for relevant literature in PubMed was carried out according to the PICOS (participants, interventions, comparisons, outcomes, and study design) references [90]. The searching keywords were not only limited to PD-1 or PD-L1, but also included specific product names and common names of related drugs. All clinical trials without a control group, meaning the single-arm clinical trial, would be excluded first. Randomized and controlled Phase III clinical trials would be prioritized, while other randomized controlled trials were considered as alternatives.

The time frame for all literature was just limited to the past ten years (August 2, 2013–August 2, 2023). The literature searching was completed by four participating authors, and the searching results would be checked by each other. In case of duplicated clinical trials, only one containing the most complete data could be used for the final analysis.

### Mirror principle pairing

To increase the similarity and minimize heterogeneity and inconsistency between groups [88, 89], the Mirror-pairing criteria were listed as follows: (1) Tumor type: Due to the significant differences among different tumor types, this is the primary criteria for the Mirror-pairing; (2) Pathological type: Tumors occurring in the same organ need to be distinguished based on specific pathological types. (3) Treatment regimen: In the combination treatment regimens including PD-1 or PD-L1 inhibitors, it is necessary to keep consistent in drug composition of the Mirror-pairing groups; (4) Treatment line; (5) Phase stage; (6) Number of participants: The two paired groups have the same order of magnitude; (7) All clinical trials data could only be used once for the best Mirror-pairing; (8) Results choice: The analyses result involving single drug regimens will be prioritized, while the analyses results of combination therapy regimens are just considered as reference; (9)Other: While the above factors have been confirmed by subgroup analysis to be not the factor causing differences in subgroup analysis results, this factor can be moderately adjusted during pairing.

### Evaluation of study quality and publication bias

Egger's test was used to test the symmetry of funnel plots [91, 92], while funnel plots and Harbor's test were used for publication bias evaluation [92, 93]. The Newcastle–Ottawa scale (NOS), recommended by Cochrane Collaboration, was used for quality assessments [94, 95]. The assessments contents were listed as follows: random sequence generation (selection bias), allocation concealment (selection bias), blinding of participants and personnel (performance bias), blinding of outcome assessment (detection bias), incomplete outcome data (attrition bias), selective reporting (reporting bias) and others. The evaluation method was to verify the original data of the reported clinical trials. *P* < 0.05 was considered to be statistically significant.

### Exposure of interest, assessment of heterogeneity, and statistical analyses

The basic characteristics of clinical trials that met the inclusion criteria were collected and summarized in a separate table. This study focused on the incidence risk of pneumonitis in all grades.

Heterogeneity was assessed by Cochrane’s Q statistic test [94, 95], including the Mantel–Haenszel method and *I*^2^ values suggested by Higgins and colleagues [90, 95]. According to the different *I*^2^ values, heterogeneity was divided into three different levels: low (*I*^2^ < 25%), medium (*I*^2^ = 25–50%), and high (*I*^2^ > 50%) [90, 95]. The software Review Manager 5.3 was used for all the following analyses. Due to the inevitable existence of intergroup heterogeneity in the real world, random effects (RE) models were used for calculating odds ratio (OR) and 95% confidence interval (CI) [96]. The fixed effects (FE) model would just be used for funnel plot evaluations. All *P* values were calculated by two sides. *P* < 0.05 was deemed to be of statistical significance. Subgroup analysis was conducted based on PD-1 or PD-L1 types. When obvious heterogeneity was discovered, more detailed subgroup analyses would be conducted based on the specific situation. If heterogeneity was considered to be mainly caused by the data itself, further processing of the data would not be carried out, and the original data analysis results would still be adopted.

## Results

### Literature search results and characteristics of identified trials

**Fig. 1:**
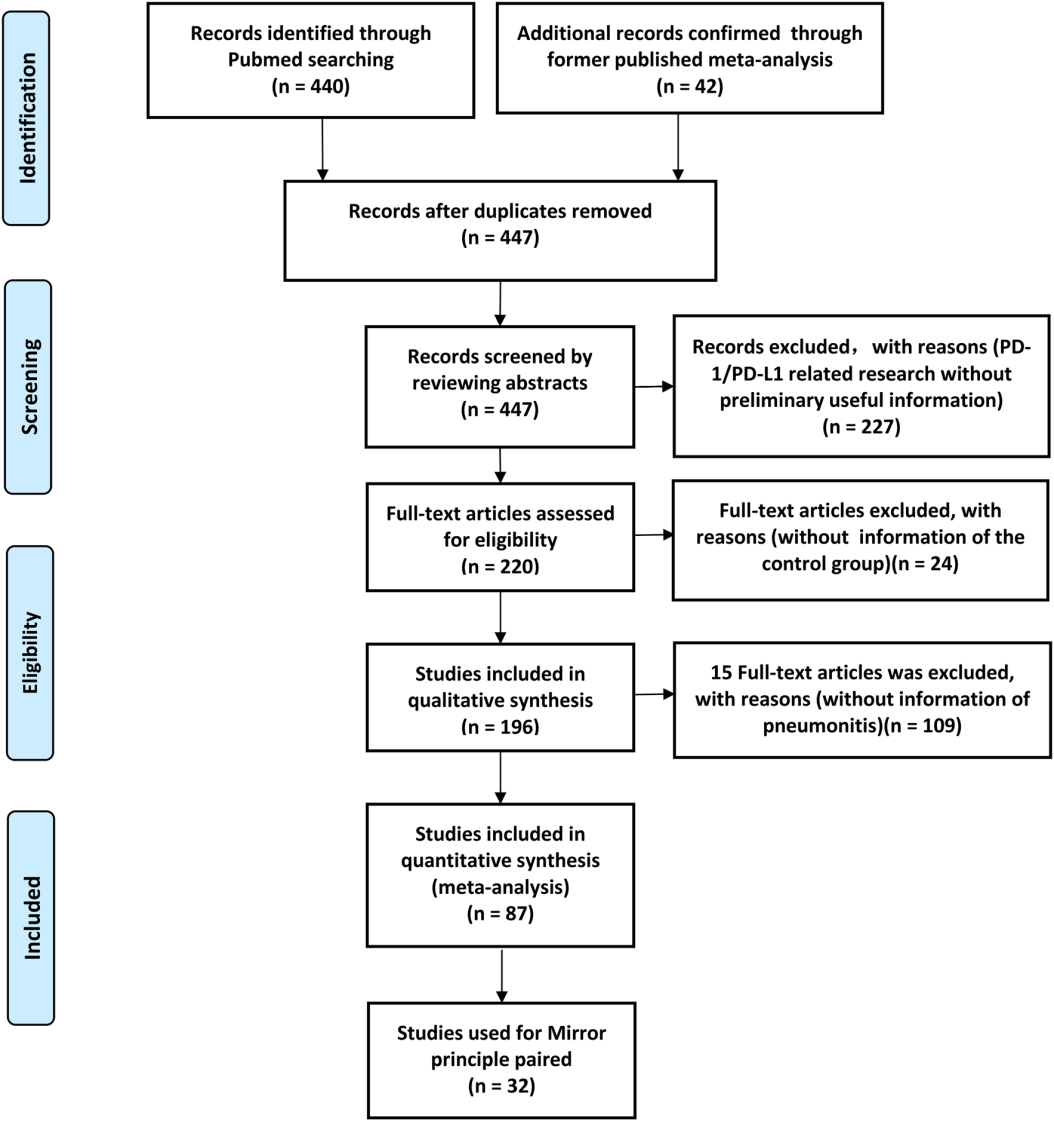
The flow diagram of the meta-analysis

Through PubMed search, 440 studies met the basic criteria. After screening and verification, 87 studies, belonging to 77 clinical trials, were screened for the final comprehensive analyses (STable 1) [1–87], and 32 were selected for the Mirror-pairing (Fig. 1; Table 1) [1, 2, 4, 6–8, 10, 12, 14, 15, 17, 19, 21–24, 36–38, 42, 43, 45, 48, 49, 51, 53, 57, 63, 71, 78, 79]. Of these, 14 clinical trials were reported more than once (KEYNOTE-010 [4, 5], KEYNOTE-042 [12, 13], IMpower110 [14, 15], KEYNOTE‑061^[17,18)^, KEYNOTE-177 [28, 29], KEYNOTE-189 [37, 41], IMpower133 [44, 47], KEYNOTE-355 [57, 62], KEYNOTE-522 [61, 63], IMpassion130 [51, 52], PACIFIC [68, 69], KEYNOTE-054 [72, 73], CheckMate227 [11, 82], CheckMate067 [83–86]). However, only one of the most completed data was used for the final comprehensive analysis or Mirror-pairing. Of all enrolled clinical trials, lung cancer (*n* = 36; STable 1) [1–16, 35–49, 65–71, 81, 82], including non-small cell lung cancer (NSCLC) and small cell lung cancer (SCLC), accounted for the highest proportion, followed by esophageal squamous cell carcinoma (ESCC) (*n* = 7) [25–27, 55, 58–60]. Seventy-one were found to be phase III [1–3, 6–34, 36–73, 75–80, 82–87], two were phase II/III [4, 5], three were phase II [6, 35, 74], and one was phase I/II [81]. Previous treatments were found in 23 clinical trials [1–9, 17, 18, 20, 21, 25–27, 30–33, 68, 69, 77, 80, 81], while PD-1 or PD-L1 inhibitors were prescribed as the first line choice in the other reports [10–16, 19, 22–24, 28, 29, 34–67, 70–76, 78, 79, 82–87]. The quality assessments of all clinical trials were finished and provided in (SFigure 1). Data evaluated as high risk would be excluded (SFigure 1).

**Table 1:** Basic characteristics of Mirror-pairing clinical trials

Mirror Group	References	NCT number	Phase	Drug name	Treatment regimen	Treatment lines	Tumor type	Involving patients
*PD-1 VS. PD-L1*								
Group 1	Reck M,et al. [10]	NCT02142738 (KEYNOTE-024)	III	Pembrolizumab (PD-1)	Pembrolizumab VS. Chemotherapy	1	Advanced NSCLC	154
	Jassem J,et al. [15]	NCT02409342 ( IMpower110)	III	Atezolizumab (PD-L1)	Atezolizumab VS. Chemotherapy		Treatment-Naive PD-L1–Selected NSCLC	286
Group 2	Borghaei H,et al. [1]	NCT01673867 (CheckMate 057)	III	Nivolumab (PD-1)	Nivolumab VS. Docetaxel	2	Advanced non-squamous NSCLC	287
	Fehrenbacher L,et al. [6]	NCT01903993 (POPLAR)	II	Atezolizumab (PD-L1)	Atezolizumab VS. Docetaxel		Previously treated NSCLC	142
Group 3	Wu YL,et al. [12]	NCT02613507 (CheckMate078)	III	Nivolumab (PD-1)	Nivolumab VS. Docetaxel	2	Advanced NSCLC	337
	Barlesi F,et al. [8]	NCT02395172 (JAVELIN Lung 200)	III	Avelumab (PD-L1)	Avelumab VS. Docetaxel		platinum-treated advanced NSCLC	393
Group 4	Brahmer J,et al. [2]	NCT01642004 (CheckMate017)	III	Nivolumab (PD-1)	Nivolumab VS. Docetaxel	2	Advanced Squamous Cell NSCLC	131
	Hida T,et al. [7]	NCT02008227 (OAK)	III	Atezolizumab (PD-L1)	Atezolizumab VS. Docetaxel		Advanced NSCLC	56
Group 5	Bajorin DF,et al. [78]	NCT02632409 (CheckMate 274)	III	Nivolumab (PD-1)	Nivolumab VS Placebo	1	muscle-invasive UC	351
	Bellmunt J,et al. [79]	NCT02450331 (IMvigor010)	III	Atezolizumab (PD-L1)	Atezolizumab vs Observation		muscle-invasive UC	390
Group 6	Powles T,et al. [21]	NCT02853305 (KEYNOTE-361)	III	Pembrolizumab (PD-1)	Pembrolizumab VS Chemotherapy	1	advanced UC	302
	Powles T,et al. [23]	NCT02516241 (DANUBE)	III	**Durvalumab** (PD-L1)	**Durvalumab VS Durvalumab + tremelimumab(CTLA-4)**		unresectable, locally advanced or metastatic UC	345
Group 7	Shitara K,et al. [17]	NCT02370498 (KEYNOTE-061)	III	Pembrolizumab (PD-1)	Pembrolizumab VS. Paclitaxel	2	Advanced GC or GEJC	294
	Moehler M,et al. [19]	NCT02625610 (JAVELINGastric 100)	III	Avelumab (PD-L1)	Avelumab VS. Chemotherapy	1	GC or GEJC	240
Group 8	Herbst RS, [4]	NCT01905657 (KEYNOTE-010)	II/III	Pembrolizumab (PD-1)	Pembrolizumab 10 mg/kg VS. Docetaxel	2	PD-L1-positive, advanced NSCLC	339
	Herbst RS, [14]	NCT02409342 (IMpower110)	III	Atezolizumab (PD-L1)	Atezolizumab VS. Chemotherapy	1	Metastatic non-squamous or squamous NSCLC	286
Group 9	Herbst RS,et al. [4]	NCT01905657 (KEYNOTE-010)	II/III	Pembrolizumab (PD-1)	Pembrolizumab 2 mg/kg VS Docetaxel	2	PD-L1-positive, advanced NSCLC	339
	Felip E,et al. [71]	NCT02486718 (IMpower010)	III	Atezolizumab (PD-L1)	Atezolizumab VS BSC	1	Resected stage IB–IIIA NSCLC	495
*PD-1 + chemotherapy VS. PD-L1 + chemotherapy*								
Group 1 +	Gandhi L,et al. [37]	NCT02578680 (KEYNOTE-189)	III	Pembrolizumab (PD-1)	Pembrolizumab + Chemotherapy VS. Chemotherapy	1	Metastatic NSCLC	405
	West H,et al. [42]	NCT02367781 (IMpower130)	III	Atezolizumab (PD-L1)	Atezolizumab + Carboplatin + nab-paclitaxel VS. Carboplatin + nab-paclitaxel	1	Metastatic non-squamous NSCLC	473
Group 2 +	Cheng Y,et al. [48]	NCT04063163 (ASTRUM-005)	III	Serplulimab (PD-1)	Serplulimab + EP VS. EP	1	ES -SCLC	389
	Paz-Ares L,et al. [45]	NCT03043872	III	Durvalumab (PD-L1)	Durvalumab + EP VS. EP	1	ES -SCLC	265
Group 3 +	Paz-Ares L,et al. [36]	NCT02775435 (KEYNOTE-407)	III	Pembrolizumab (PD-1)	Pembrolizumab + Carboplatin + Paclitaxel VS. Carboplatin + Paclitaxel	1	untreated metastatic, squamous NSCLC	278
	Jotte R,et al. [49]	NCT02367794 (IMpower131)	III	Atezolizumab (PD-L1)	Atezolizumab + Carboplatin + Paclitaxel VS. Carboplatin + nab-paclitaxel	1	Advanced Squamous NSCLC	332
Group 4 +	Zhou C,et al. [38]	NCT03629925 (ORIENT-12)	III	Sintilimab (PD-1)	Sintilimab + GP VS. GP	1	Advanced or Metastatic Squamous NSCLC	179
	Zhou C. [43]	NCT03789604 (GEMSTONE-302)	III	Sugemalimab (PD-L1)	Sugemalimab + GP VS. GP	1	metastatic NSCLC	320
Group 5 +	Powles T,et al. [22]	NCT02853305 (KEYNOTE-361)	III	Pembrolizumab (PD-1)	Pembrolizumab + Chemotherapy VS. Chemotherapy	1	Advanced UC	349
	Galsky MD,et al. [24]	NCT02807636 (IMvigor130)	III	Atezolizumab (PD-L1)	Atezolizumab + Chemotherapy VS. Chemotherapy	1	Locally advanced or metastatic UC	453
Group 6 +	Cortes J,et al. [57]	NCT02819518 (KEYNOTE-355)	III	Pembrolizumab (PD-1)	Pembrolizumab + Chemotherapy VS. Chemotherapy	1	Untreated locally recurrent inoperable or metastatic TNBC	562
	Emens LA,et al. [51]	NCT02425891 (IMpassion130)	III	Atezolizumab (PD-L1)	Atezolizumab + nab-paclitaxel VS. nab-paclitaxel	1	Unresectable locally advanced, or metastatic TNBC	460
Group 7 +	Schmid P,et al. [63]	NCT03036488 (KEYNOTE-522)	III	Pembrolizumab (PD-1)	Pembrolizumab + Chemotherapy VS. Chemotherapy	1	Early TNBC	784
	Mittendorf EA,et al. [53]	NCT03197935 (IMpassion031)	III	Atezolizumab (PD-L1)	Atezolizumab + Chemotherapy VS. Chemotherapy	1	Early Stage TNBC	164

PD-L1 = Programmed Cell Death-1; PD-L1 = Programmed Cell Death Ligand 1; CTLA-4 = Cytotoxic T lymphocyte associate protein-4; OSCC = Oesophageal Squamous Cell Carcinoma; UC = Urothelial Cancer; NSCLC = Non-Small Cell Lung Cancer, HNSCC = Head and Neck Squaous Cell Carcinoma, GC/GEJC = Gastric or Gastro-oesophageal Junction Cancer, TNBC = Triple-negative Breast Cancer, SCLC = Small Cell Lung Cancer, HCC = Hepatocellular Carcinoma, RCC = Renal Cell Carcinoma, CRC = Colorectal Cancer; MPM = malignant pleural mesothelioma

According to the composition of treatment regimens, all enrolled clinical trials were divided into five groups for the comprehensive analyses. The specific groupings were listed as follows: Group A (PD-1/PD-L1 VS. Chemotherapy) [1–34], Group B (PD-1/PD-L1 + Chemotherapy VS. Chemotherapy) [35–67], Group C (PD-1/PD-L1 VS. Placebo) [68–80], Group D (PD-1/PD-L1 VS. PD-1/PD-L1 + CTLA-4) [11, 23, 81–86], and Group E (PD-1/PD-L1 VS.PD-1/PD-L1 + Chemotherapy) [19, 21, 24, 34, 82].

### Results of mirror pairing

After comprehensive analyses and comparison of all enrolled clinical trials, 32 clinical trials were paired according to the Mirror-pairing principle and divided into 16 groups [1, 2, 4, 6–8, 10, 12, 14, 15, 17, 19, 21–24, 36–38, 42, 43, 45, 48, 49, 51, 53, 57, 63, 71, 78, 79], including 9 pairs for the PD-1 versus PD-L1 group and 7 pairs for the chemotherapy combination group (Table 1). Among these Mirror-pairing clinical trials, NSCLC (*n* = 17) accounted for the highest proportion [1, 2, 4, 6–8, 10, 12, 14, 15, 36–38, 42, 43, 49, 71], followed by urothelial carcinoma (UC) (*n* = 6) [21–24, 78, 79].

### Risk of pneumonitis

**Fig. 2:**
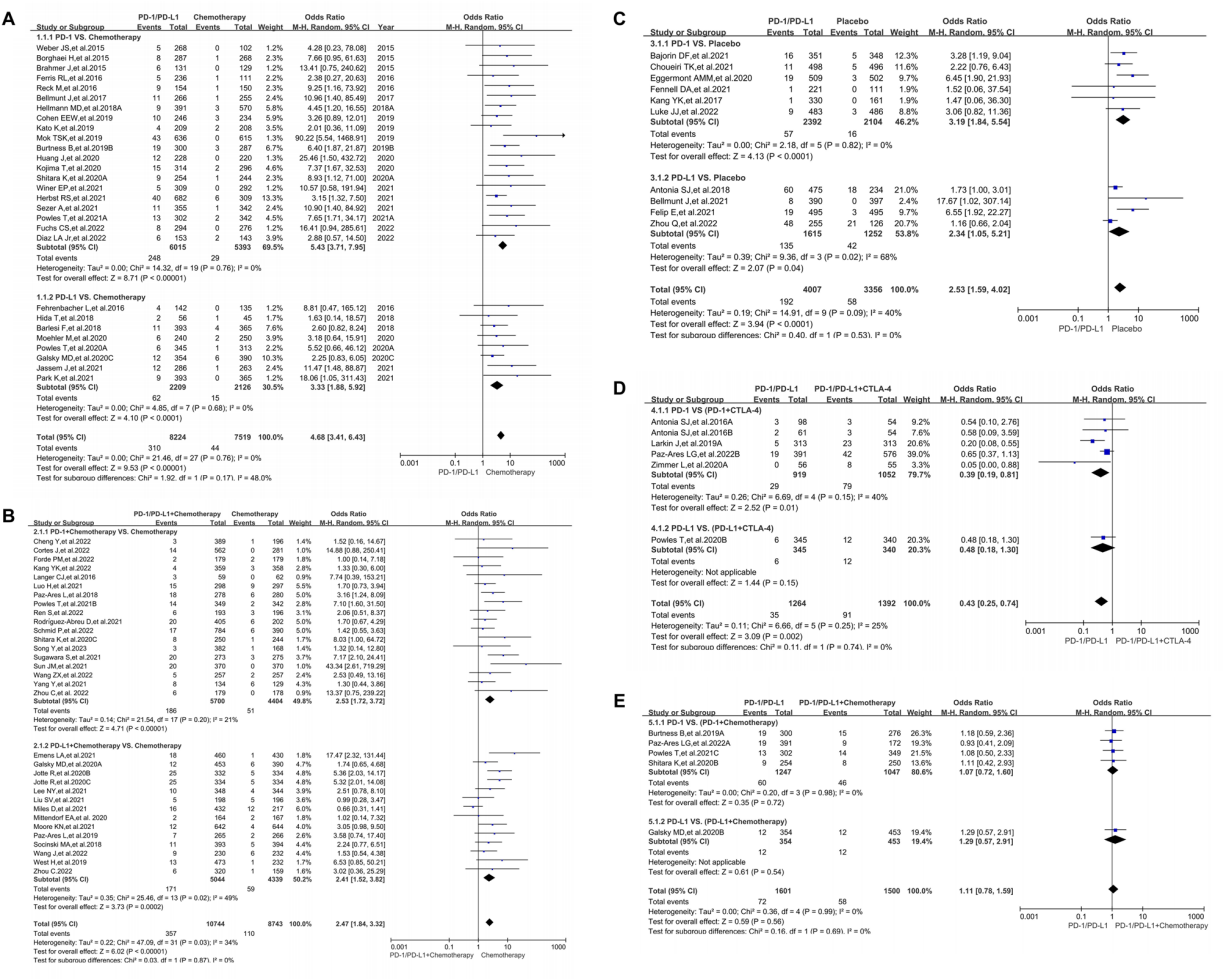
Forest blots of the analysis results for different groups. **A** The OR of pneumonitis for all-grade checked using the random effect (RE) model in Group A (PD-1/PD-L1 VS. Chemotherapy): Subgroup analyses were carried out according to the types of immune checkpoint inhibitors (PD-1 or PD-L1). **B** The OR of pneumonitis for all-grade checked using the random effect (RE) model in Group B (PD-1/PD-L1 + Chemotherapy VS. Chemotherapy): Subgroup analyses were carried out according to the types of immune checkpoint inhibitors (PD-1 or PD-L1). **C** The OR of pneumonitis for all-grade checked using the random effect (RE) model in Group C (PD-1/PD-L1 VS. Placebo): Subgroup analyses were carried out according to the types of immune checkpoint inhibitors (PD-1 or PD-L1). **D** The OR of pneumonitis for all-grade checked using the random effect (RE) model in Group D (PD-1/PD-L1 VS. PD-1/PD-L1 + CTLA-4): Subgroup analyses were carried out according to the types of immune checkpoint inhibitors (PD-1 or PD-L1). **E** The OR of pneumonitis for all-grade checked using the random effect (RE) model in Group E (PD-1/PD-L1 VS. PD-1/PD-L1 + Chemotherapy): Subgroup analyses were carried out according to the types of immune checkpoint inhibitors (PD-1 or PD-L1)

Compared with chemotherapy (PD-1/PD-L1 VS. Chemotherapy), PD-1/PD-L1 inhibitors significantly increased the risk of immune-related pneumonitis (OR = 4.68, 95%CI [3.41, 6.43], *I*^2^ = 0%, *Z* = 9.53 (*P* < 0.00001); Fig. 2A), and similar risk trend was also found in the subgroup analysis [1–3, 5–11, 13, 15–27, 29–34]. The PD-1 subgroup (OR = 5.43) had a higher risk of developing immune-related pneumonitis than the PD-L1 subgroup (OR = 3.33; Fig. 2A), while there was no statistical significance (*P* = 0.17). No heterogeneity was found in the above results (*I*^2^ = 0%). No significant publication bias was found through the corresponding funnel plots (SFigure 2A). Further subgroup analysis based on different tumor types revealed a higher risk of pneumonitis in the PD-1 subgroup of different tumor types (SFigure 4), especially for the Gastric or Gastro-oesophageal Junction Cancer (GC/GEJC) and UC subgroups.

When PD-1 or PD-L1 inhibitors combined with chemotherapy were compared with chemotherapy alone (PD-1/PD-L1 + Chemotherapy VS. Chemotherapy), the risk of immune-related pneumonitis was also significantly increased (OR = 2.47, 95%CI [1.84, 3.32], *I*^2^ = 34%, *Z* = 6.02 (*P* < 0.00001); Fig. 2B) [20, 21, 23, 35, 36, 38–43, 45–51, 53–56, 58–60, 62–67]. A slightly higher risk of developing pneumonitis could be found in the PD-1 subgroup (2.53 vs. 2.41; Fig. 2B). For moderate heterogeneity (*I*^2^ = 34%), further subgroup analysis indicated that it might be caused by 3 Triple-negative Breast Cancer (TNBC) clinical trials (IMpassion130, IMpassion031, IMpassion131; SFigure 5) [51, 53, 56]. No significant publication bias was found through the corresponding funnel plots (SFigure 2B).

When the control group was placebo but chemotherapy (PD-1/PD-L1 VS. Placebo), the incidence risk of pneumonitis was also increased by PD-1/PD-L1 inhibitors (OR = 2.53, 95%CI [1.59, 4.02], *I*^2^ = 40%, *Z* = 3.94(*P* < 0.0001); Fig. 2C) [69–73, 75–80]. Similar to the above, subgroup analysis indicated that the PD-1 subgroup presented a higher risk of developing pneumonitis (3.19 VS. 2.34; Fig. 2C). For moderate heterogeneity (*I*^2^ = 40%), further subgroup analysis indicated that it might be caused by three NSCLC clinical trials (PACIFIC, GEMSTONE-301, IMpower010; SFigure 6) [69–71]. No significant publication bias was found through the corresponding funnel plots (SFigure 2C).

Compared with the combination of PD-1/PD-L1 and CTLA-4 (PD-1/PD-L1 VS. PD-1/PD-L1 + CTLA-4), the impact of PD-1/PD-L1 on the risk of pneumonitis was weaker than that of the control group (OR = 0.43, 95%CI [0.25, 0.74], *I*^2^ = 25%, *Z* = 3.09 (*P* = 0.002); Fig. 2D) [23, 74, 81, 82, 86]. For moderate heterogeneity (*I*^2^ = 25%), subgroup analysis suggested that heterogeneity might originate from the data themselves (SFigure 7) [23, 74, 81, 82, 86]. No significant publication bias was found through the corresponding funnel plots (SFigure 2D).

Compared with PD-1/PD-L1 in combination with chemotherapy (PD-1/PD-L1 VS. PD-1/PD-L1 + Chemotherapy), the risk of pneumonitis was not significantly increased (OR = 1.11, 95%CI [0.78, 1.59], *I*^2^ = 0%, *Z* = 0.59 (*P* = 0.56); Fig. 2E) [19, 21, 24, 34, 82]. No significant publication bias was found through the corresponding funnel plot (SFigure 2E).

### Risk of pneumonitis for mirror-pairing

The basic characteristics of 16 Mirror pairings were provided in (Table 1) [1, 2, 4, 6–8, 10, 12, 14, 15, 17, 19, 21–24, 36–38, 42, 43, 45, 48, 49, 51, 53, 57, 63, 71, 78, 79]. Through the Mirror-pairing (*n* = 9) analysis of PD-1 versus PD-L1, it indicated that PD-1 had a much more significant impact on the risk of pneumonitis (OR = 1.46, 95%CI [1.08, 1.98], *I*^2^ = 0%, *Z* = 2.47 (*P* = 0.01); Fig. S3A) [1, 2, 4, 6–8, 10, 12, 14, 15, 17, 19, 21, 23, 71, 78, 79]. This risk trend was obviously evident in the UC subgroup (OR = 2.39, 95%CI [1.25, 4.57], *I*^2^ = 0%, *Z* = 2.64 (*P* = 0.008); Fig. S3A) [21, 23, 78, 79]. No heterogeneity was found. No significant publication bias was found through the corresponding funnel plots (SFigure 3A).

When chemotherapy was added to both experimental and control groups (*n* = 7), the difference became no longer significant (OR = 1.05, 95%CI [0.68, 1.60], *I*^2^ = 38%, *Z* = 0.21 (*P* = 0.84); Fig. Fig. S3B) [22, 24, 36–38, 42, 43, 45, 48, 49, 51, 53, 57, 63]. For moderate heterogeneity (*I*^2^ = 38%), subgroup analysis suggested that heterogeneity might originate from the NSCLC subgroup (Fig. Fig. S3B) [36–38, 42, 43, 45, 48, 49]. No significant publication bias was found through the corresponding funnel plot (SFigure 3B).

## Discussion

With the increasing use of PD-1/PD-L1 inhibitors in clinical practice, the complex and diverse forms of immune-related toxic side effects are increasingly reported and valued by clinical doctors [1–87]. Pneumonitis, as an important clinical event of pulmonary toxicities, requires rapid identification and management. Once suspected, the scope of differential diagnosis between infectious and vegetative processes might make the physician's diagnostic process challenging [97]. A comprehensive assessment of the incidence risk of immune-related pneumonitis would have important guiding significance for physicians. However, due to the lack of clinical trials comparing PD-1 and PD-L1 head to head, it was difficult to determine the differences in risk of pneumonitis occurrence between the two. To address this dilemma, this study was designed [88, 89].

A literature searching was conducted according to PRISMA guidelines and PICOS principles [90], and a total of 77 clinical trial data were collected (Fig. 1; STable 1) [1–87]. Seventy-seven clinical trials were taken into account for a more comprehensive and detailed analysis by grouping in more ways, which increased the possibility of obtaining more Mirror pairings and reduced the possibility of bias due to insufficient data [88, 89]. We carefully reviewed the data of all enrolled clinical trials and conducted a comprehensive systematic evaluation of random sequence generation (selection bias), allocation consideration (selection bias), blinding of participants and personnel (performance bias), blinding of outcome assessment (detection bias), incomplete outcome data (attrition bias), selective reporting (reporting bias), and others. After the comprehensive evaluation, data with any kind of high risk biases would be excluded, and only the high-quality and complete clinical trial data were retained, ensuring the reliability and authenticity of our analyses results (SFigure 1; STable 1). The previous Mirror-pairing principles had been improved^[88.89]^, which would make the pairing much more accurate. After a detailed analysis of clinical trials using the Mirror-pairing principle, 16 Mirror pairings were obtained, which was the largest number of PD-1/PD-L1 related Mirror pairings first reported so far (Table 1; SFigure 3) [1, 2, 4, 6–8, 10, 12, 14, 15, 17, 19, 21–24, 36–38, 42, 43, 45, 48, 49, 51, 53, 57, 63, 71, 78, 79]. These further strengthened the innovation of our research.

Through analysis, regardless of whether the control group was chemotherapy or placebo (Fig. 2A and C), PD-1/PD-L1 inhibitors increased the risk of immune-related pneumonitis [1–3, 5–11, 13, 15–27, 29–34, 69–73, 75–80]. Subgroup analysis indicated that the PD-1 subgroup presented a higher risk of developing pneumonitis (Fig. 2A and C) [1–3, 5–11, 13, 15–27, 29–34, 69–73, 75–80]. Although subgroup analyses could not draw statistically significant conclusions, PD-1 might cause a higher risk trend for pneumonitis (Fig. 2A and C) [1–3, 5–11, 13, 15–27, 29–34, 69–73, 75–80], which laid the foundation for the following Mirror-pairing meta-analysis.

Compared with chemotherapy, the PD-1/PD-L1 inhibitors played a much more important role in increasing the risk of immune-related pneumonitis (Fig. 2B and E) [19–24, 34–36, 38–43, 45–51, 53–56, 58–60, 62–67, 82]. When PD-1/PD-L1 inhibitors were combined with CTLA-4, this effect was obviously evident (Fig. 2D) [23, 74, 81, 82, 86]. Based on the above analyses, we concluded that PD-1/PD-L1 inhibitors increased the risk of pneumonitis; Furthermore, it seemed that PD-1 inhibitors had a higher risk of causing pneumonitis (Fig. 2) [1–87], which further enhanced the necessity of conducting Mirror-pairing analysis.

When using the Mirror-pairing for comparing PD-1 with PD-L1, the risk of pneumonitis caused by PD-1 was significantly higher than that of the PD-L1 group (OR = 1.46, 95%CI [1.08, 1.98], *I*^2^ = 0%, *Z* = 2.47 (*P* = 0.01); Fig. S3A) [1, 2, 4, 6–8, 10, 12, 14, 15, 17, 19, 21, 23, 71, 78, 79], which the difference was statistically significant. When chemotherapy was incorporated into the Mirror-pairing, this difference became no longer statistically significant (OR = 1.05, 95%CI [0.68, 1.60], *I*^2^ = 38%, *Z* = 0.21 (*P* = 0.84); Fig. S3B) [22, 24, 36–38, 42, 43, 45, 48, 49, 51, 53, 57, 63]. In the previous subgroup analyses (Fig. 2A and B), similar results could also be found after the addition of chemotherapy. Therefore, we concluded that chemotherapy might induce excessive heterogeneity and inconsistency and desalinate the true differences between PD-1 and PD-L1 (Figs. 2A, B, and S3A). When there were fewer interfering factors, whether it was indirect subgroup analysis (Fig. 2A) or the Mirror-pairing analysis (Fig. S3A), the conclusions drawn were consistent, which further confirmed the practicality and feasibility of this improved Mirror-pairing analysis method [88, 89]. The difference in the risk of pneumonitis between PD-1 and PD-L1 was evaluated using the Mirror-pairing meta-analysis, accompanied by improvements in the Mirror-pairing method, which indicated a better innovation. This comparative method solves the dilemma of lacking head-to-head clinical trials of PD-1 versus PD-L1.

Due to the inevitable existence of intergroup heterogeneity in the real world, RE models were used for OR and 95% CI calculations [96]. Although no highly heterogeneous results were found, we conducted sufficient subgroup analyses and speculated on the source of the corresponding heterogeneity (SFigure 4, SFigure 5, SFigure 6, and SFigure 7) [1–87]. There were no data found that affected the analysis results. Furthermore, no significant bias was found through the corresponding funnel plot (SFigure 2 and SFigure 3), which confirmed the authenticity and reliability of the above analysis results.

Based on the subgroup analysis results (Fig. S3A, SFigure 4, SFigure 5, and SFigure 6), we found that the risk of pneumonitis in UC patients receiving PD-1 inhibitors was the highest among all tumor types. This meant that special attention should be paid to the risk of immune-related pneumonitis for PD-1 inhibitor use in UC patients.

By comparing the subgroup analysis results of the PRISMA meta-analysis with the results of the Mirror-pairing analysis, we found that the risk trend of the analysis results was basically consistent, while the analysis results of the Mirror-pairing seemed to be much more sensitive (Figs. 2A,2C; S3A). It indicated that when mild differences in subgroup analysis was found, the Mirror-pairing analysis could be conducted to clarify the significance of these differences. Furthermore, this would be beneficial for clinicians to determine the choice of drugs (PD-1 or PD-L1) based on the degree of toxicities, as well as whether PD-1 was needed to be replaced by PD-L1.

## Conclusions

Both PD-1 and PD-L1 inhibitors increased the risk of immune-related pneumonitis, while the risk of PD-1-induced pneumonitis was significantly higher than that of PD-L1 inhibitors.

### The limitations of the study

The Mirror-pairing analysis is an indirect paired comparison of existing clinical trials while minimizing heterogeneity. Its reliability still needs to be validated with more head-to-head clinical trial data in the real world.

### Supplementary Information

Below is the link to the electronic supplementary material.

S Table 1: Basic characteristics of all enrolled clinical trials. (DOCX 72 KB)

S Figure 1: Risk of bias summary: review authors' judgements about each risk of bias item for each included study. (TIF 1698 KB)

S Figure 2: Funnel plots of the analysis results for different groups. A: The OR of pneumonitis for all-grade checked using the fixed effect (FE) model in Group A (PD-1/PD-L1 VS. Chemotherapy): Subgroup analyses were carried out according to the types of immune checkpoint inhibitors (PD-1 or PD-L1). B: The OR of pneumonitis for all-grade checked using the fixed effect (FE) model in Group B (PD-1/PD-L1+Chemotherapy VS. Chemotherapy): Subgroup analyses were carried out according to the types of immune checkpoint inhibitors (PD-1 or PD-L1). C: The OR of pneumonitis for all-grade checked using the fixed effect (FE) model in Group C (PD-1/PD-L1 VS. Placebo): Subgroup analyses were carried out according to the types of immune checkpoint inhibitors (PD-1 or PD-L1). D: The OR of pneumonitis for all-grade checked using the fixed effect (FE) model in Group D (PD-1/PD-L1 VS. PD-1/PD-L1+CTLA-4): Subgroup analyses were carried out according to the types of immune checkpoint inhibitors (PD-1 or PD-L1). E: The OR of pneumonitis for all-grade checked using the fixed effect (FE) model in Group E (PD-1/PD-L1 VS. PD-1/PD-L1+Chemotherapy): Subgroup analyses were carried out according to the types of immune checkpoint inhibitors (PD-1 or PD-L1). (TIF 2042 KB)

S Figure 3: Funnel plots of comparison in Mirror-pairing clinical trials. A: The OR of pneumonitis for all grades was checked using the fixed effect (FE) model (PD-1 VS. PD-L1). Subgroup analyses were carried out according to the tumor types. B: The OR of pneumonitis for all grades was checked using the fixed effect (FE) model (PD-1+Chemotherapy VS. PD-L1+Chemotherapy). Subgroup analyses were carried out according to the tumor types. (TIF 2023 KB)

S Figure 4: Forest blots of the subgroup analysis in Group A (PD-1/PD-L1 VS. Chemotherapy): The OR of pneumonitis for all-grade checked using the random effect (RE) model: Subgroup analyses were carried out according to the tumor types and PD-1/PD-L1. (TIF 2856 KB)

S Figure 5: Forest blots of the subgroup analysis in Group B (PD-1/PD-L1+Chemotherapy VS. Chemotherapy): The OR of pneumonitis for all-grade checked using the random effect (RE) model: Subgroup analyses were carried out according to the tumor types and PD-1/PD-L1. (TIF 2913 KB)

S Figure 6: Forest blots of the subgroup analysis in Group C (PD-1/PD-L1 VS. Placebo): The OR of pneumonitis for all-grade checked using the random effect (RE) model: Subgroup analyses were carried out according to the tumor types and PD-1/PD-L1. (TIF 2504 KB)

S Figure 7: Forest blots of the subgroup analysis in Group D (PD-1/PD-L1 VS. PD-1/PD-L1+CTLA-4): The OR of pneumonitis for all-grade checked using the random effect (RE) model: Subgroup analyses were carried out according to the tumor types and PD-1/PD-L1. (TIF 1597 KB)

## Abbreviations

CI: Confidence interval
CnP: Carboplatin + Nab-paclitaxel
CP: Carboplatin + Paclitaxel
CRC: Colorectal cancer
CTLA-4: Cytotoxic T lymphocyte associate protein-4
EC: Etoposide + Carboplatin
EP: Etoposide + Platinum
ESCC: Oesophageal squamous cell carcinoma
GC/GEJC: Gastric or gastro-oesophageal junction cancer
GC/GP: Carboplatin/Cisplatin + Gemcitabine
HCC: Hepatocellular carcinoma
HNSCC: Head and neck squamous cell carcinoma
HR: Hazard ratios
NOS: Newcastle–Ottawa scale
NSCLC: Non-small cell lung cancer
OR: Odds ratio
PD-1: Programmed cell death-1
PD-L1: Programmed cell death ligand 1
PF: Cisplatin + Fluorouracil
PICOS: Participants, interventions, comparisons, outcomes, and study design
PRISMA: Preferred reporting items for systematic reviews and meta-analyses
RCC: Renal cell carcinoma
RE: Random effect
SCLC: Small cell lung cancer
TNBC: Triple-negative breast cancer
UC: Urothelial cancer
UC: Urothelial cancer
