# Supplementary material for: Differences in the risk of immune-related pneumonitis between PD-1 and PD-L1 inhibitors: a meta-analysis according to the new mirror-principle and PRISMA guidelines
Source: Cancer Immunol Immunother. 2024 Jul 2;73(9):162. doi: 10.1007/s00262-024-03736-z (PMC11219650; doi:10.1007/s00262-024-03736-z)
Supplement: Supplementary file 1 — S Table 1: Basic characteristics of all enrolled clinical trials. (DOCX 72 KB) [file 262_2024_3736_MOESM1_ESM.docx]

| **NO** | **Reference** | **NCT number** | **Phase** | **Drug name** | **Treatment regimen** | **Tumor types** | **Previous treatment** | **Involving patients** | **Immune related Pneumonitis** |
| --- | --- | --- | --- | --- | --- | --- | --- | --- | --- |
| 1 | Borghaei H,et al.2015^(1)^ | NCT01673867  (CheckMate057) | Ⅲ | Nivolumab  (PD-1) | Nivolumab VS. Docetaxel | NSCLC | Yes | 555 | 9 |
| 2 | Brahmer J,et al.2015^(2)^ | NCT01642004  (CheckMate017) | Ⅲ | Nivolumab  (PD-1) | Nivolumab VS. Docetaxel | NSCLC | Yes | 260 | 6 |
| 3 | Wu YL,et al.2019^(3)^ | NCT02613507  (CheckMate078) | Ⅲ | Nivolumab  (PD-1) | Nivolumab VS. Docetaxel | NSCLC | Yes | 493 | 15 |
| 4 | Herbst RS,et al.2016A^(4)^ | NCT01905657  (KEYNOTE-01) | Ⅱ/Ⅲ | Pembrolizumab  (PD-1) | Pembrolizumab 2mg/kg VS. Pembrolizumab 10mg/kg | NSCLC | Yes | 682 | 31 |
|  | Herbst RS,et al.2016B^(4)^ |  |  |  | Pembrolizumab 2mg/kg VS. Docetaxel |  |  | 648 | 22 |
|  | Herbst RS,et al.2016C^(4)^ |  |  |  | Pembrolizumab 10mg/kg VS. Docetaxel |  |  | 652 | 21 |
| 5 | Herbst RS,et al.2021^(5)^ | NCT01905657  (KEYNOTE-01) | Ⅱ/Ⅲ | Pembrolizumab  (PD-1) | Pembrolizumab VS. Docetaxel | NSCLC | Yes | 991 | 49 |
| 6 | Fehrenbacher L,et al.2016^(6)^ | NCT01903993  (POPLAR) | Ⅱ | Atezolizumab  (PD-L1) | Atezolizumab VS. Docetaxel | NSCLC | Yes | 277 | 4 |
| 7 | Hida T,et al.2018^(7)^ | NCT02008227  (OAK ) | Ⅲ | Atezolizumab  (PD-L1) | Atezolizumab VS. Docetaxel | NSCLC | Yes | 101 | 3 |
| 8 | Barlesi F,et al. 2018^(8)^ | NCT02395172  (JAVELIN Lung 200) | Ⅲ | Avelumab  (PD-L1) | Avelumab VS. Docetaxel | NSCLC | Yes | 758 | 15 |
| 9 | Park K,et al.2021^(9)^ | NCT02576574  (JAVELIN Lung 200) | III | Avelumab  (PD-L1) | Avelumab VS Docetaxel | NSCLC | Yes | 758 | 9 |
| 10 | Reck M,et al.2016^(10)^ | NCT02142738  (KEYNOTE-024) | Ⅲ | Pembrolizumab  (PD-1) | Pembrolizumab VS. Platinum-based Chemotherapy | NSCLC | No | 304 | 10 |
| 11 | Hellmann MD,et al.2018A^(11)^ | NCT02477826  (CheckMate227) | Ⅲ | Nivolumab  (PD-1) | Nivolumab VS. Nivolumab+ Ipilimumab | NSCLC | No | 967 | 31 |
|  | Hellmann MD,et al.2018B^(11)^ |  |  |  | Nivolumab+ Ipilimumab VS. Platinum doublet Chemotherapy |  |  | 1146 | 25 |
|  | Hellmann MD,et al.2018C^(11)^ |  |  |  | Nivolumab VS. Platinum doublet Chemotherapy |  |  | 961 | 12 |
| 12 | Wu YL,et al.2021^(12)^ | NCT02220894  (KEYNOTE-042) | III | Pembrolizumab  (PD-1) | Pembrolizumab VS. Carboplatin + Paclitaxel or Pemetrexed | NSCLC | No | 253 | 10 |
| 13 | Mok TSK,et al.2019^(13)^ |  |  |  |  |  |  | 1251 | 56 |
| 14 | Herbst RS,et al.2020^(14)^ | NCT02409342  (IMpower110) | III | Atezolizumab  (PD-L1) | Atezolizumab VS. Platinum-based chemotherapy | NSCLC | No | 549 | 12 |
| 15 | Jassem J,et al.2021^(15)^ |  |  |  |  |  |  | 549 | 13 |
| 16 | Sezer A,et al.2021^(16)^ | NCT03088540  (EMPOWER-Lung 1) | III | Cemiplimab  (PD-L1) | Cemiplimab VS Chemotherapy | NSCLC | No | 697 | 7 |
| 17 | Shitara K,et al.2018^(17)^ | NCT02370498  (KEYNOTE-061) | III | Pembrolizumab  (PD-1) | Pembrolizumab VS. Paclitaxel | GC/GEJC | Yes | 570 | 8 |
| 18 | Fuchs CS,et al.2022^(18)^ |  |  |  |  |  |  |  |  |
| 19 | Moehler M,et al.2020^(19)^ | NCT02625610  (JAVELIN  Gastric 100) | III | Avelumab  (PD-L1) | Avelumab VS. Chemotherapy | GC or GEJC | No | 490 | 8 |
| 20 | Shitara K,et al.2020A^(20)^ | NCT02494583  (KEYNOTE-062) | Ⅲ | Pembrolizumab  (PD-1) | Pembrolizumab VS Pembrolizumab + Chemotherapy (Cisplatin +Fluorouracil or Capecitabine ) | GC/GEJC | Yes | 504 | 17 |
|  | Shitara K,et al.2020B^(20)^ |  |  |  | Pembrolizumab VS Chemotherapy (Cisplatin +Fluorouracil or Capecitabine ) |  |  | 498 | 10 |
|  | Shitara K,et al.2020C^(20)^ |  |  |  | Pembrolizumab+Chemotherapy (Cisplatin + Fluorouracil or Capecitabine ) VS Chemotherapy (Cisplatin + Fluorouracil or Capecitabine ) |  |  | 494 | 9 |
| 21 | Bellmunt J,et al.2017^(21)^ | NCT02256436  (KEYNOTE-045) | Ⅲ | Pembrolizumab  (PD-1) | Pembrolizumab VS. (Paclitaxel, Docetaxel, or Vinflunine) | UC | Yes | 521 | 12 |
| 22 | Powles T,et al.2021A^(22)^ | NCT02853305  (KEYNOTE-36) | III | Pembrolizumab  (PD-1) | Pembrolizumab + Chemotherapy VS. Pembrolizumab | UC | No | 651 | 27 |
|  | Powles T,et al.2021B^(22)^ |  |  |  | Pembrolizumab + Chemotherapy VS. Chemotherapy |  |  | 691 | 16 |
|  | Powles T,et al.2021C^(22)^ |  |  |  | Pembrolizumab VS. Chemotherapy |  |  | 644 | 15 |
| 23 | Powles T,et al.2020A^(23)^ | NCT02516241  (DANUBE) | Ⅲ | Durvalumab  (PD-L1) | Durvalumab VS. Durvalumab + Tremelimumab | UC | No | 685 | 17 |
|  | Powles T,et al.2020B^(23)^ |  |  |  | Durvalumab VS. GP |  |  | 658 | 7 |
|  | Powles T,et al.2020C^(23)^ |  |  |  | Durvalumab + Tremelimumab VS. GP |  |  | 653 | 12 |
| 24 | Galsky MD,et al.2020A^(24)^ | NCT02807636  (IMvigor130) | Ⅲ | Atezolizumab  (PD-L1) | Atezolizumab VS. Atezolizumab + GP | UC | No | 807 | 24 |
|  | Galsky MD,et al.2020B^(24)^ |  |  |  | Atezolizumab + GP VS. GP |  |  | 843 | 18 |
|  | Galsky MD,et al.2020C^(24)^ |  |  |  | Atezolizumab VS. GP |  |  | 744 | 18 |
| 25 | Kojima T,et al.2020^(25)^ | KEYNOTE-181 | Ⅲ | Pembrolizumab  (PD-1) | Pembrolizumab VS. Chemotherapy (Paclitaxel, Docetaxel, or Irinotecan) | ESCC | Yes | 610 | 17 |
| 26 | Kato K,et al.2019^(26)^ | NCT02569242  (ATTRACTION-3) | Ⅲ | Nivolumab  (PD-1) | Nivolumab VS. (Paclitaxel or Docetaxel ) | ESCC | Yes | 417 | 6 |
| 27 | Huang J,et al.2020^(27)^ | NCT03099382  (ESCORT) | III | Camrelizumab  (PD-1) | Camrelizumab VS. Chemotherapy | ESCC | Yes | 448 | 12 |
| 28 | André T,et al.2020^(28)^ | NCT0256300  (KEYNOTE-177) | Ⅲ | Pembrolizumab  (PD-1) | Pembrolizumab VS. Chemotherapy | CRC | No | 296 | 7 |
| 29 | Diaz LA Jr,et al.2022^(29)^ | NCT02563002  (KEYNOTE-177) | III | Pembrolizumab  (PD-1) | Pembrolizumab VS. Chemotherapy | Colorectal Cancer | No | 296 | 8 |
| 30 | Winer EP,et al.2020^(30)^ | NCT02555657  (KEYNOTE-119) | Ⅲ | Pembrolizumab  (PD-1) | Pembrolizumab VS. Chemotherapy (Capecitabine, Eribulin, Gemcitabine, or Vinorelbine) | TNBC | Yes | 601 | 6 |
| 31 | Weber JS,et al.2015^(31)^ | NCT01721746  (CheckMate037) | Ⅲ | Nivolumab  (PD-1) | Nivolumab VS. (Dacarbazine or Paclitaxel + Carboplatin) | Melanoma | Yes | 370 | 5 |
| 32 | Cohen EEW,et al.2019^(32)^ | NCT02252042  (KEYNOTE-040) | Ⅲ | Pembrolizumab  (PD-1) | Pembrolizumab VS. (Methotrexate, Docetaxel or Cetuximab) | HNSCC | Yes | 480 | 13 |
| 33 | Ferris RL,et al.2016^(33)^ | NCT02105636  (CheckMate141) | Ⅲ | Nivolumab  (PD-1) | Nivolumab VS. (Methotrexate, Docetaxel, or Cetuximab) | HNSCC | Yes | 347 | 6 |
| 34 | Burtness B,et al.2019A^(34)^ | NCT02358031  (KEYNOTE-048) | Ⅲ | Pembrolizumab  (PD-1) | Pembrolizumab VS. Pembrolizumab + Chemotherapy | HNSCC | No | 576 | 34 |
|  | Burtness B,et al.2019B^(34)^ |  |  |  | Pembrolizumab VS. Cetuximab + Chemotherapy |  |  | 587 | 22 |
|  | Burtness B,et al.2019C^(34)^ |  |  |  | Pembrolizumab + Chemotherapy VS.Cetuximab + Chemotherapy |  |  | 563 | 18 |
| 35 | Langer CJ,et al.2016^(35)^ | NCT02039674  (KEYNOTE-021) | Ⅱ | Pembrolizumab  (PD-1) | Pembrolizumab + Carboplatin + Pemetrexed VS. Carboplatin + Pemetrexed | NSCLC | No | 121 | 3 |
| 36 | Paz-Ares L,et al.2018^(36)^ | NCT02775435  (KEYNOTE-407) | Ⅲ | Pembrolizumab  (PD-1) | Pembrolizumab + CP/CnP VS. CP/CnP | NSCLC | No | 558 | 24 |
| 37 | Gandhi L,et al.2018^(37)^ | NCT02578680  (KEYNOTE-189) | Ⅲ | Pembrolizumab  (PD-1) | Pembrolizumab + Pemetrexed + platinum-based drug VS. Pemetrexed + platinum-based drug | NSCLC | No | 607 | 23 |
| 38 | Zhou C,et al.2022^(38)^ | NCT03629925  (ORIENT-12) | III | Sintilimab  (PD-1) | Sintilimab + GP VS. GP | Squamous NSCLC | No | 357 | 6 |
| 39 | Forde PM,et al.2022^(39)^ | NCT02998528  (CheckMate 816) | III | Nivolumab  (PD-1) | Nivolumab + Chemotherapy VS. Chemotherapy | NSCLC | No | 358 | 4 |
| 40 | Ren S,et al.2022^(40)^ | NCT03668496  (CameL-Sq) | III | Camrelizumab  (PD-1) | Camrelizumab + Chemotherapy VS. Chemotherapy | Squamous NSCLC | No | 389 | 9 |
| 41 | Rodríguez-Abreu D,et al.2021^(41)^ | NCT02578680  (KEYNOTE-189) | III | Pembrolizumab  (PD-1) | Pembrolizumab + Chemotherapy VS. Chemotherapy | NSCLC | No | 607 | 26 |
| 42 | West H,et al.2019^(42)^ | NCT02367781  (IMpower130) | Ⅲ | Atezolizumab  (PD-L1) | Atezolizumab + CnP VS. CnP | NSCLC | No | 705 | 34 |
| 43 | Zhou C,et al.2022^(43)^ | NCT03789604  (GEMSTONE-302) | III | Sugemalimab  (PD-L1) | Sugemalimab + Chemotherapy VS. Chemotherapy | Metastatic NSCLC | No | 379 | 7 |
| 44 | Horn L,et al.2018^(44)^ | NCT02763579  (IMpower133) | Ⅲ | Atezolizumab  (PD-L1) | Atezolizumab + EC VS. EC | SCLC | No | 394 | 9 |
| 45 | Paz-Ares L,et al.2019^(45)^ | NCT03043872  (CASPIAN) | Ⅲ | Durvalumab  (PD-L1) | Durvalumab + EP VS. EP | SCLC | No | 531 | 9 |
| 46. | Wang J,et al.2022^(46)^ | NCT03711305  (CAPSTONE-1) | III | Adebrelimab  (PD-L1) | Adebrelimab + Chemotherapy VS. Chemotherapy | ES - SCLC | No | 462 | 15 |
| 47 | Liu SV,et al.2021^(47)^ | NCT02763579  (IMpower133) | III | Atezolizumab  (PD-L1) | Atezolizumab + CP/ET VS. CP/ET | ES -SCLC | No | 394 | 10 |
| 48 | Cheng Y,et al.2022^(48)^ | NCT04063163  (ASTRUM-005) | III | Serplulimab  (PD-1) | Serplulimab + Chemotherapy VS. Chemotherapy | ES -SCLC | No | 585 | 4 |
| 49 | Jotte R,et al.2020A^(49)^ | NCT02367794  (IMpower131) | Ⅲ | Atezolizumab  (PD-L1) | Atezolizumab + CP VS. Atezolizumab + CnP | NSCLC | No | 666 | 50 |
|  | Jotte R,et al.2020B^(49)^ |  |  |  | Atezolizumab + CP VS. CnP |  |  | 666 | 30 |
|  | Jotte R,et al.2020C^(49)^ |  |  |  | Atezolizumab + CnP VS. CnP |  |  | 668 | 30 |
| 50 | Kang YK,et al.2017^(50)^ | NCT02267343  (ATTRACTION-2) | Ⅲ | Nivolumab  (PD-1) | Nivolumab VS. Placebo | GC/GEJC | No | 491 | 1 |
| 51 | Emens LA,et al.2021^(51)^ | NCT02425891  (IMpassion130) | III | Atezolizumab  (PD-L1) | Atezolizumab + Nab-paclitaxel VS. Nab-paclitaxel | TNBC | No | 890 | 19 |
| 52 | Schmid P,et al.2018^(52)^ |  |  |  |  |  |  | 890 | 15 |
| 53 | Mittendorf EA,et al.2020^(53)^ | NCT03197935  (IMpassion031) | Ⅲ | Atezolizumab  (PD-L1) | Atezolizumab + Chemotherapy VS. Chemotherapy | TNBC | No | 331 | 4 |
| 54 | Yang Y,et al.2021^(54)^ | NCT03707509  (CAPTAIN-1st) | III | Camrelizumab  (PD-1) | Camrelizumab + GP VS. GP | Nasopharyngeal Carcinoma | No | 263 | 14 |
| 55 | Luo H,et al.2021^(55)^ | NCT03691090  (ESCORT-1st) | III | Camrelizumab  (PD-1) | Camrelizumab + Chemotherapy VS Chemotherapy | ESCC | No | 595 | 24 |
| 56 | Miles D,et al.2021^(56)^ | NCT03125902  (IMpassion131) | III | Atezolizumab  (PD-L1) | Atezolizumab + Paclitaxel VS. Paclitaxel | TNBC | No | 649 | 28 |
| 57 | Cortes J,et al.2020^(57)^ | NCT02819518  (KEYNOTE-355) | Ⅲ | Pembrolizumab  (PD-1) | Pembrolizumab + Chemotherapy VS. Chemotherapy(Nab-paclitaxel; Paclitaxel; or Gemcitabine plus Carboplatin) | TNBC | No | 843 | 14 |
| 58 | Sun JM,et al.2021^(58)^ | NCT03189719  (KEYNOTE-590) | III | Pembrolizumab  (PD-1) | Pembrolizumab + PF VS. PF | ESCC | No | 740 | 20 |
| 59 | Song Y,et al.2023^(59)^ | NCT03958890 | III | Serplulimab  (PD-1) | Serplulimab + Chemotherapy VS. Chemotherapy | ESCC | No | 550 | 4 |
| 60 | Wang ZX,et al.2022^(60)^ | NCT03829969  (JUPITER-06) | III | Toripalimab  (PD-1) | Toripalimab + TP VS. TP | ESCC | No | 514 | 7 |
| 61 | Schmid P,et al.2020^(61)^ | NCT03036488  (KEYNOTE-522) | Ⅲ | Pembrolizumab  (PD-1) | Pembrolizumab + CP VS. CP | TNBC | No | 1170 | 15 |
| 62 | Cortes J,et al.2022^(62)^ | NCT02819518  (KEYNOTE-355) | III | Pembrolizumab  (PD-1) | Pembrolizumab + Chemotherapy VS. Chemotherapy | TNBC | No | 843 | 14 |
| 63 | Schmid P,et al.2022^(63)^ | NCT03036488  (KEYNOTE-522) | III | Pembrolizumab  (PD-1) | Pembrolizumab + Chemotherapy VS. Chemotherapy | TNBC | No | 1174 | 23 |
| 64 | Lee NY,et al.2021^(64)^ | NCT02952586  (JAVELIN Head and Neck 100 trial) | Ⅲ | Avelumab  (PD-L1) | Avelumab + Chemoradiotherapy VS. Chemoradiotherapy | HNSCC | No | 692 | 5 |
| 65 | Socinski MA,et al.2018^(65)^ | NCT02366143  (IMpower150) | Ⅲ | Atezolizumab  (PD-L1) | Atezolizumab + Bevacizumab + CP VS. Bevacizumab + CP | NSCLC | No | 787 | 16 |
| 66 | Moore KN,et al.2021^(66)^ | NCT03038100 | III | Atezolizumab  (PD-L1) | Atezolizumab + CP + Bevacizumab VS CP + Bevacizumab | Ovarian Cancer | No | 1286 | 16 |
| 67 | Sugawara S,et al.2021^(67)^ | NCT03117049  (ONO-4538-52/TASUKI-52) | III | Nivolumab  (PD-1) | Nivolumab + CP + Bevacizumab VS. CP + Bevacizumab | NSCLC | No | 548 | 23 |
| 68 | Antonia SJ,et al.2017^(68)^ | NCT02125461  (PACIFIC) | Ⅲ | Durvalumab  (PD-L1) | Durvalumab VS. Placebo | NSCLC | Yes | 709 | 67 |
| 69 | Antonia SJ,et al.2018^(69)^ |  |  |  |  |  |  |  | 78 |
| 70 | Zhou Q,et al.2022^(70)^ | NCT03728556  (GEMSTONE-30) | III | Sugemalimab  (PD-L1) | Sugemalimab VS. Placebo | NSCLC | No | 381 | 69 |
| 71 | Felip E,et al.2021^(71)^ | NCT02486718  (IMpower010) | III | NCT02486718  (IMpower010) | Atezolizumab VS. BSC | NSCLC | No | 990 | 22 |
| 72 | Eggermont AMM,et al.2018^(72)^ | NCT02362594  (KEYNOTE-054) | Ⅲ | Pembrolizumab  (PD-1) | Pembrolizumab VS. Placebo | Melanoma | No | 1011 | 20 |
| 73 | Eggermont AMM,et al.2020^(73)^ |  |  |  |  |  |  |  | 22 |
| 74 | Zimmer L,et al.2020A^(74)^ | NCT02523313  (IMMUNED ) | Ⅱ | Nivolumab  (PD-1) | Nivolumab VS. Nivolumab + Ipilimumab | Melanoma | No | 111 | 8 |
|  | Zimmer L,et al.2020B^(74)^ |  |  |  | Nivolumab + Ipilimumab VS. Placebo |  |  | 106 | 8 |
|  | Zimmer L,et al.2020C^(74)^ |  |  |  | Nivolumab VS. Placebo |  |  | 107 | 0 |
| 75 | Luke JJ,et al.2022^(75)^ | NCT03553836  (KEYNOTE-716) | III | Pembrolizumab  (PD-1) | Pembrolizumab VS. Placebo | Melanoma | No | 969 | 12 |
| 76 | Choueiri TK,et al.2021^(76)^ | NCT03142334  (KEYNOTE-564) | III | Pembrolizumab  (PD-1) | Pembrolizumab VS Placebo | RCC | No | 994 | 16 |
| 77 | Kang YK,et al.2022^(77)^ | NCT02746796  (ATTRACTION-4) | III | Nivolumab  (PD-1) | Nivolumab + Chemotherapy VS. Chemotherapy | GC or GEJC | Yes | 717 | 7 |
| 78 | Bajorin DF,et al.2021^(78)^ | NCT02632409  (CheckMate 274) | III | Nivolumab  (PD-1) | Nivolumab VS. Placebo | UC | No | 699 | 21 |
| 79 | Bellmunt J,et al.2021^(79)^ | NCT02450331  (IMvigor010) | III | Atezolizumab  (PD-L1) | Atezolizumab VS. Observation | UC | No | 787 | 8 |
| 80 | Fennell DA,et al.2021^(80)^ | NCT03063450  (CONFIRM) | III | Nivolumab  (PD-1) | Nivolumab VS. Placebo | Mesothelioma | Yes | 332 | 1 |
| 81 | Antonia SJ,et al.2016A^(81)^ | NCT01928394  (CheckMate032) | Ⅰ/Ⅱ | Nivolumab  (PD-1) | Nivolumab 3mg/kg VS.Nivolumab 1mg/kg + Ipilimumab 3mg/kg | SCLC | Yes | 159 | 5 |
|  | Antonia SJ,et al.2016B^(81)^ |  |  |  | Nivolumab 3mg/kg VS.Nivolumab 3 mg/kg + Ipilimumab1 mg/kg |  |  | 152 | 6 |
|  | Antonia SJ,et al.2016C^(81)^ |  |  |  | Nivolumab 1mg/kg + Ipilimumab 3mg/kg VS. Nivolumab 3 mg/kg + Ipilimumab  1 mg/kg |  |  | 115 | 5 |
| 82 | Paz-Ares LG, 2022A^(82)^ | NCT02477826  (CheckMate 227 PART 1) | III | Nivolumab  (PD-1) | Nivolumab VS. Nivolumab + Chemotherapy | NSCLC | No | 1139 | 70 |
|  | Paz-Ares LG, 2022B^(82)^ |  |  |  | Nivolumab VS. Nivolumab + Ipilimumab |  |  |  |  |
|  | Paz-Ares LG, 2022C^(82)^ |  |  |  | Nivolumab + Ipilimumab VS. Nivolumab + Chemotherapy |  |  |  |  |
| 83 | Larkin J,et al.2015A^(83)^ | NCT01844505  (CheckMate067) | Ⅲ | Nivolumab  (PD-1) | Nivolumab VS. Nivolumab + Ipilimumab | Melanoma | No | 626 | 24 |
|  | Larkin J,et al.2015B^(83)^ |  |  |  | Nivolumab + Ipilimumab VS. Ipilimumab |  |  | 624 | N/A |
|  | Larkin J,et al.2015C^(83)^ |  |  |  | Nivolumab VS. Ipilimumab |  |  | 624 | N/A |
| 84 | Wolchok JD,et al.2017A^(84)^ |  |  |  | Nivolumab VS. Nivolumab + Ipilimumab |  |  | 626 | 27 |
|  | Wolchok JD,et al.2017B^(84)^ |  |  |  | Nivolumab + Ipilimumab VS. Ipilimumab |  |  | 624 | 27 |
|  | Wolchok JD,et al.2017C^(84)^ |  |  |  | Nivolumab VS. Ipilimumab |  |  | 624 | 10 |
| 85 | Hodi FS,et al.2018A^(85)^ |  |  |  | Nivolumab VS. Nivolumab + Ipilimumab |  |  | 626 | 28 |
|  | Hodi FS,et al.2018B^(85)^ |  |  |  | Nivolumab + Ipilimumab VS. Ipilimumab |  |  | 624 | 28 |
|  | Hodi FS,et al.2018C^(85)^ |  |  |  | Nivolumab VS. Ipilimumab |  |  | 624 | 10 |
| 86 | Larkin J,et al.2019A^(86)^ |  |  |  | Nivolumab VS. Nivolumab + Ipilimumab |  |  | 626 | 28 |
|  | Larkin J,et al.2019B^(86)^ |  |  |  | Nivolumab + Ipilimumab VS. Ipilimumab |  |  | 624 | 28 |
|  | Larkin J,et al.2019C^(86)^ |  |  |  | Nivolumab VS. Ipilimumab |  |  | 624 | 10 |
| 87 | Ascierto PA et al,2020^(87)^ | NCT02388906  (CheckMate 238) | Ⅲ | Nivolumab  (PD-1) | Nivolumab VS Ipilimumab | Melanoma | No | 905 | 1 |

PD-1 = Programmed Cell Death-1, PD-L1 = Programmed Cell Death Ligand 1; CTLA-4 = Cytotoxic T lymphocyte associate protein-4; ESCC = Oesophageal Squamous Cell Carcinoma; UC = Urothelial Cancer, NSCLC = Non-Small Cell Lung Cancer; HNSCC = Head and Neck Squamous Cell Carcinoma; GC/GEJC = Gastric or Gastro-oesophageal Junction Cancer; TNBC = Triple-negative Breast Cancer; SCLC = Small Cell Lung Cancer; HCC = Hepatocellular Carcinoma; RCC = Renal Cell Carcinoma; CRC= Colorectal Cancer; MPM=malignant pleural mesothelioma; GP = Carboplatin/Cisplatin+ Gemcitabine; CP = Carboplatin + Paclitaxel; CnP = Carboplatin + nab-paclitaxel; EC = Etoposide + Carboplatin; EP = Etoposide + Platinum; PF = Cisplatin + Fluorouracil.
