# Supplementary figures and images for: Differences in the risk of immune-related pneumonitis between PD-1 and PD-L1 inhibitors: a meta-analysis according to the new mirror-principle and PRISMA guidelines
Source: Cancer Immunol Immunother. 2024 Jul 2;73(9):162. doi: 10.1007/s00262-024-03736-z (PMC11219650; doi:10.1007/s00262-024-03736-z)

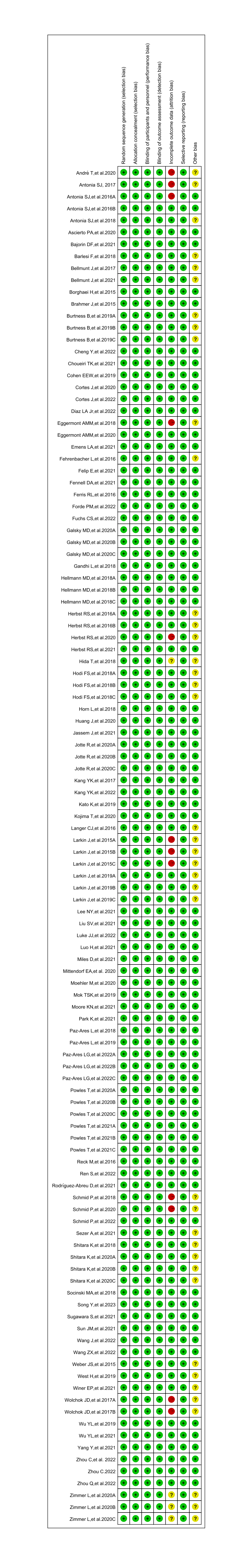

Supplement: Supplementary file 2 — S Figure 1: Risk of bias summary: review authors' judgements about each risk of bias item for each included study. (TIF 1698 KB) [file 262_2024_3736_MOESM2_ESM.tif]

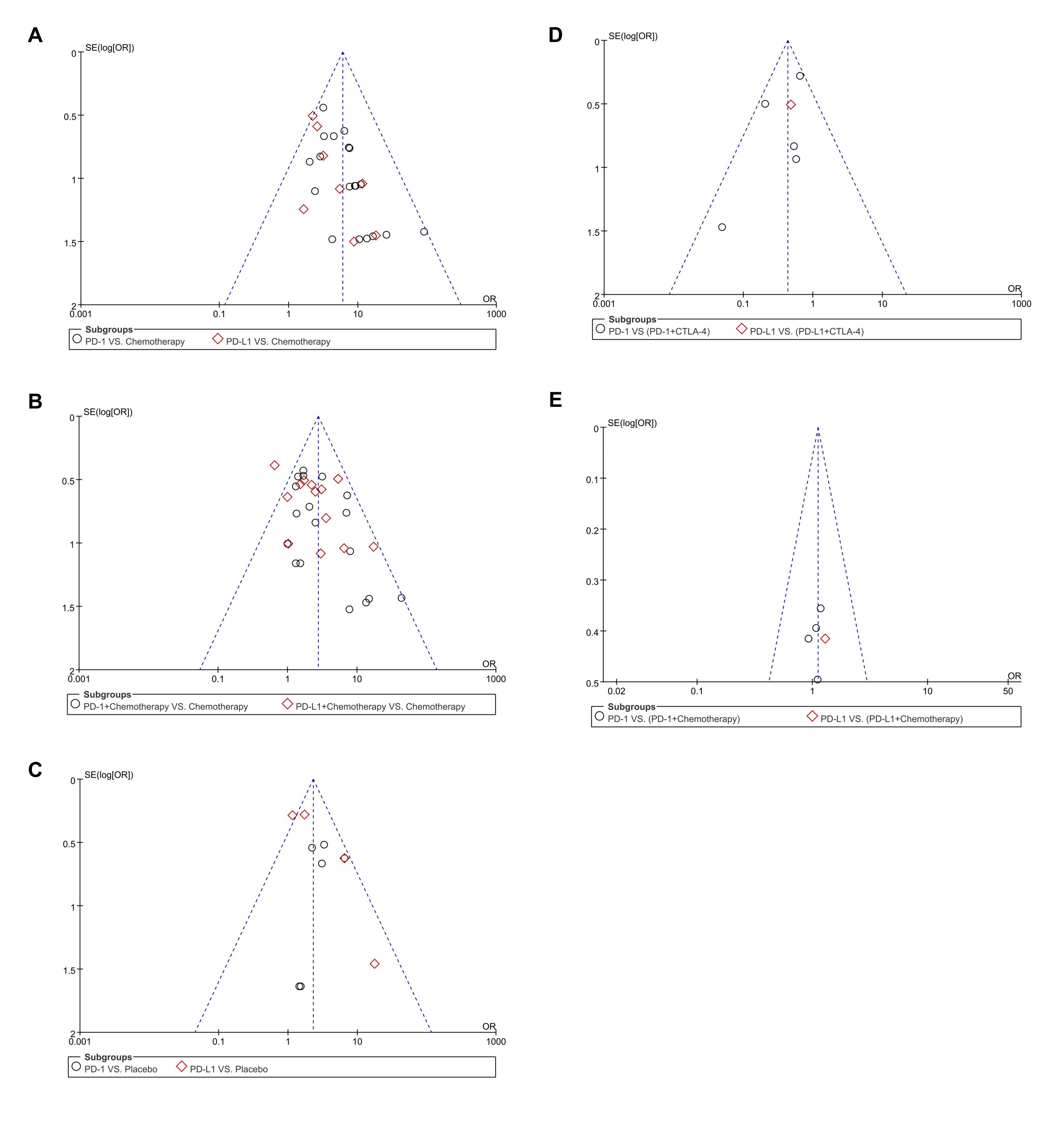

Supplement: Supplementary file 3 — S Figure 2: Funnel plots of the analysis results for different groups. A: The OR of pneumonitis for all-grade checked using the fixed effect (FE) model in Group A (PD-1/PD-L1 VS. Chemotherapy): Subgroup analyses were carried out according to the types of immune checkpoint inhibitors (PD-1 or PD-L1). B: The OR of pneumonitis for all-grade checked using the fixed effect (FE) model in Group B (PD-1/PD-L1+Chemotherapy VS. Chemotherapy): Subgroup analyses were carried out according to the types of immune checkpoint inhibitors (PD-1 or PD-L1). C: The OR of pneumonitis for all-grade checked using the fixed effect (FE) model in Group C (PD-1/PD-L1 VS. Placebo): Subgroup analyses were carried out according to the types of immune checkpoint inhibitors (PD-1 or PD-L1). D: The OR of pneumonitis for all-grade checked using the fixed effect (FE) model in Group D (PD-1/PD-L1 VS. PD-1/PD-L1+CTLA-4): Subgroup analyses were carried out according to the types of immune checkpoint inhibitors (PD-1 or PD-L1). E: The OR of pneumonitis for all-grade checked using the fixed effect (FE) model in Group E (PD-1/PD-L1 VS. PD-1/PD-L1+Chemotherapy): Subgroup analyses were carried out according to the types of immune checkpoint inhibitors (PD-1 or PD-L1). (TIF 2042 KB) [file 262_2024_3736_MOESM3_ESM.tif]

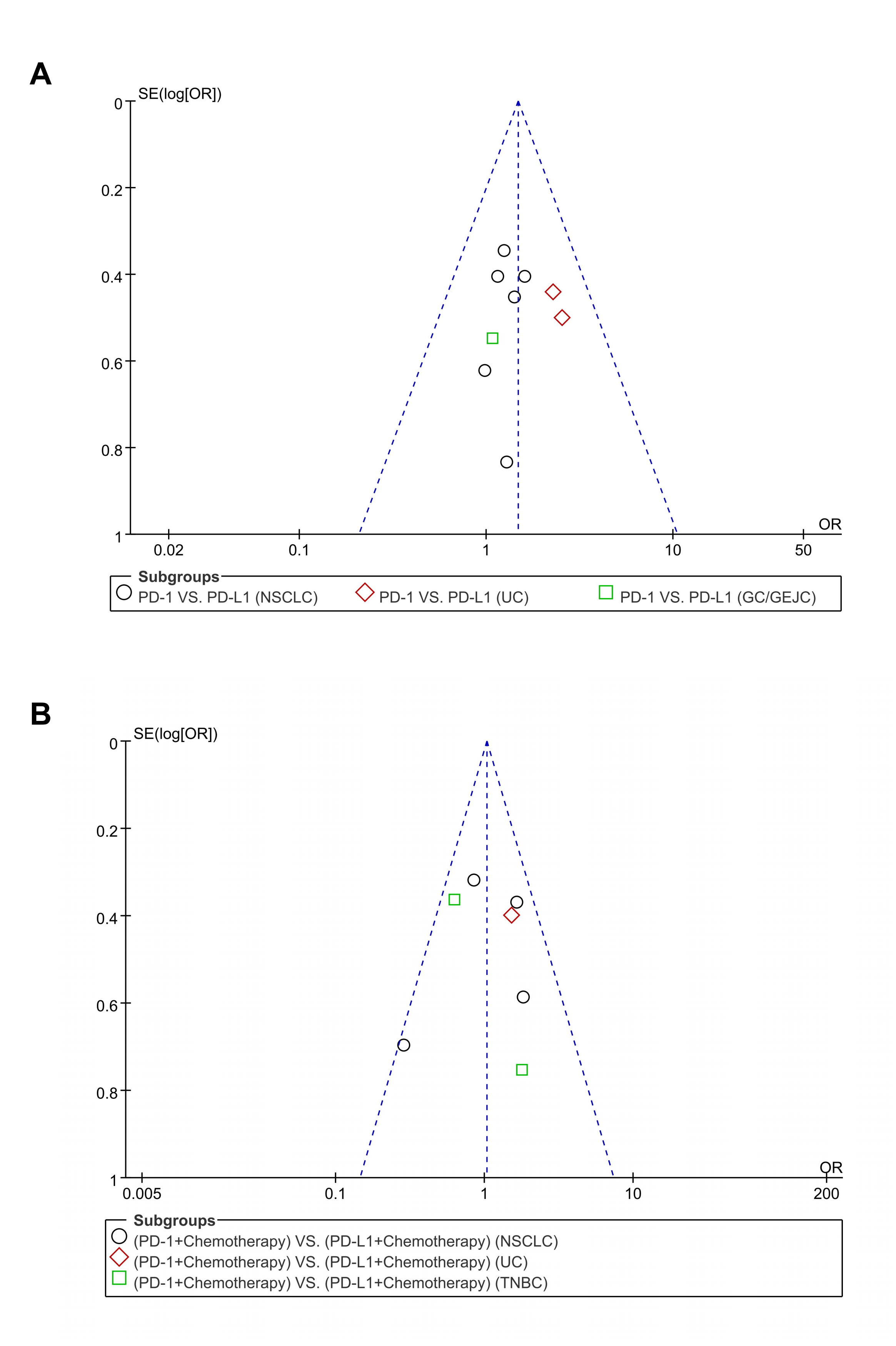

Supplement: Supplementary file 4 — S Figure 3: Funnel plots of comparison in Mirror-pairing clinical trials. A: The OR of pneumonitis for all grades was checked using the fixed effect (FE) model (PD-1 VS. PD-L1). Subgroup analyses were carried out according to the tumor types. B: The OR of pneumonitis for all grades was checked using the fixed effect (FE) model (PD-1+Chemotherapy VS. PD-L1+Chemotherapy). Subgroup analyses were carried out according to the tumor types. (TIF 2023 KB) [file 262_2024_3736_MOESM4_ESM.tif]

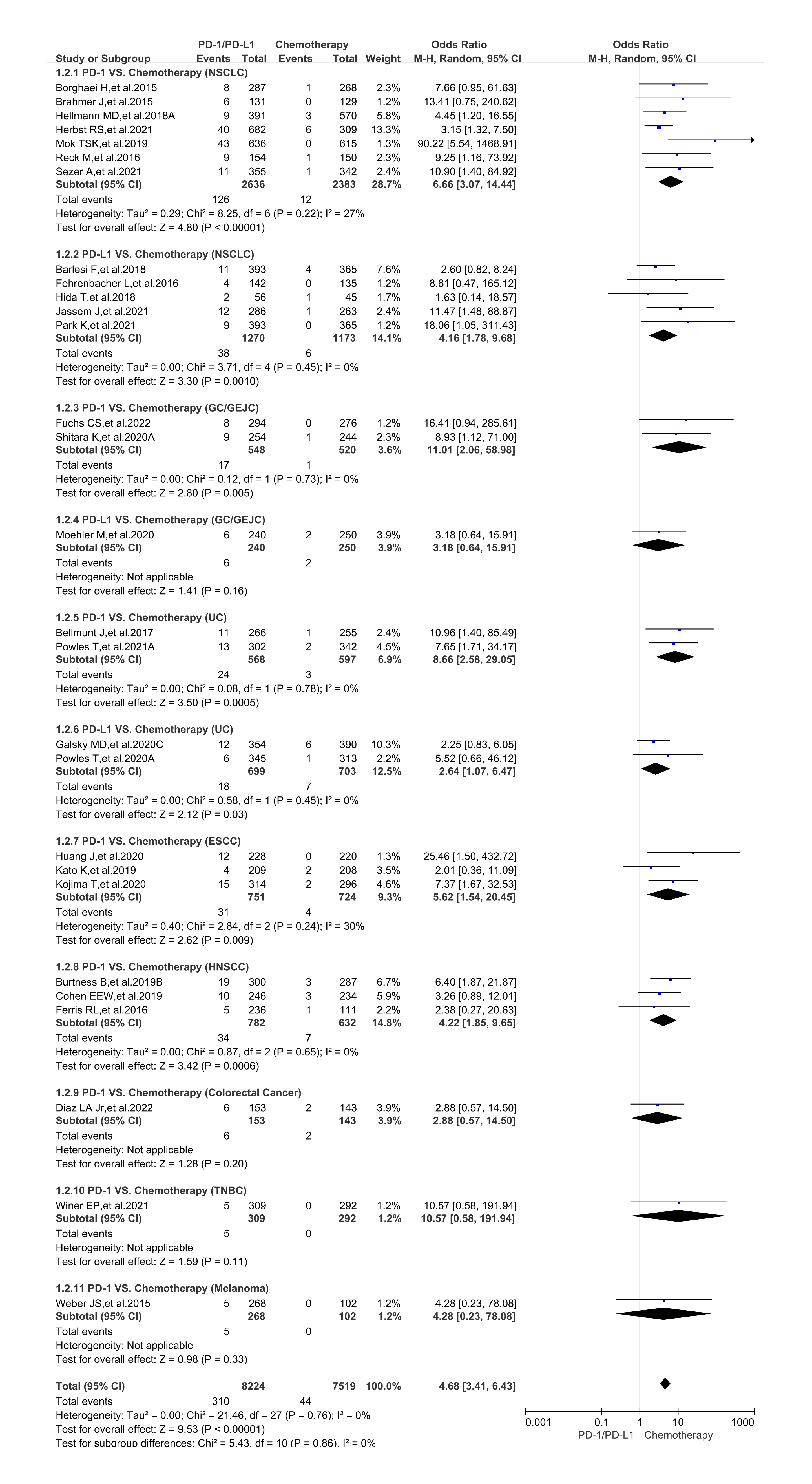

Supplement: Supplementary file 5 — S Figure 4: Forest blots of the subgroup analysis in Group A (PD-1/PD-L1 VS. Chemotherapy): The OR of pneumonitis for all-grade checked using the random effect (RE) model: Subgroup analyses were carried out according to the tumor types and PD-1/PD-L1. (TIF 2856 KB) [file 262_2024_3736_MOESM5_ESM.tif]

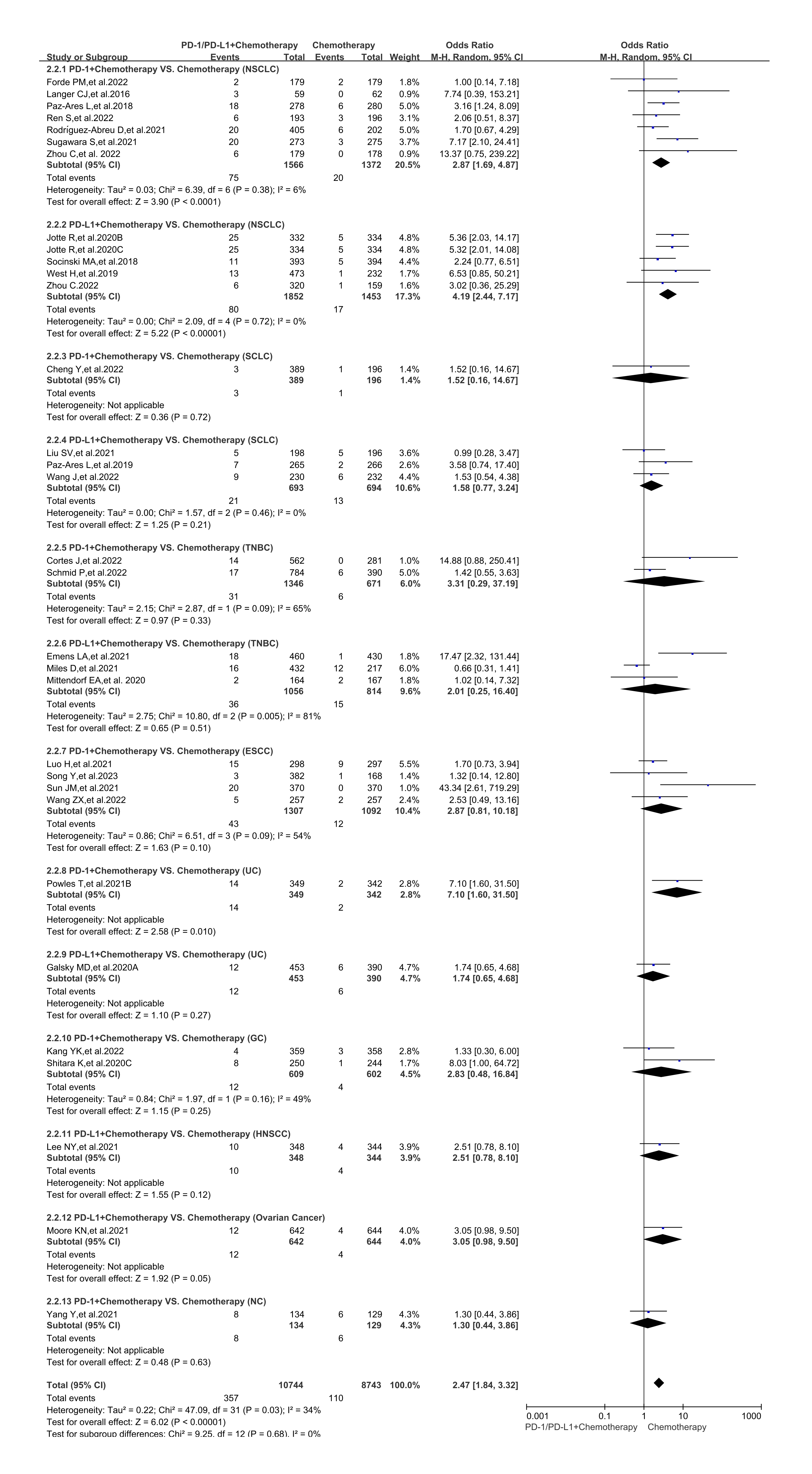

Supplement: Supplementary file 6 — S Figure 5: Forest blots of the subgroup analysis in Group B (PD-1/PD-L1+Chemotherapy VS. Chemotherapy): The OR of pneumonitis for all-grade checked using the random effect (RE) model: Subgroup analyses were carried out according to the tumor types and PD-1/PD-L1. (TIF 2913 KB) [file 262_2024_3736_MOESM6_ESM.tif]

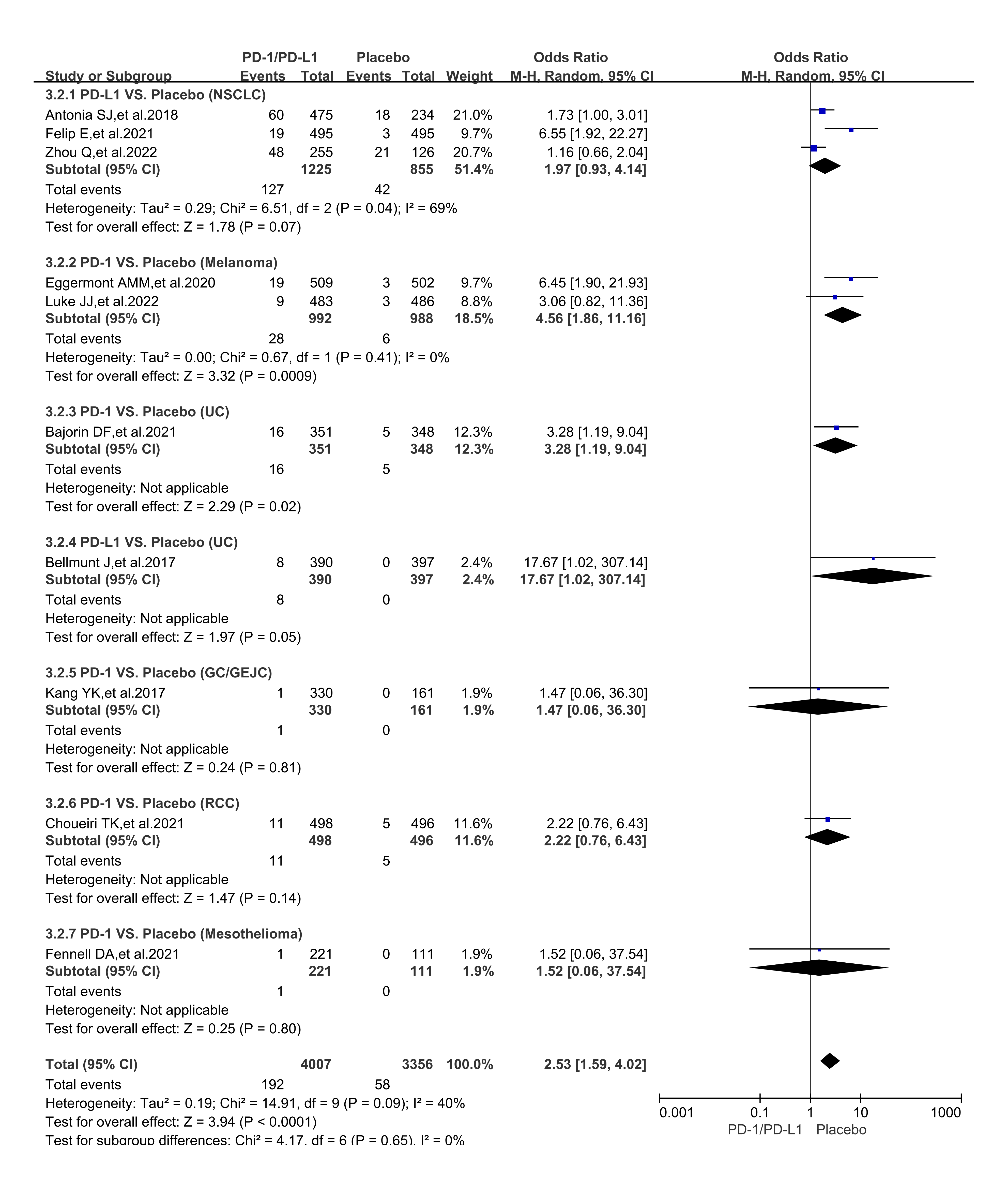

Supplement: Supplementary file 7 — S Figure 6: Forest blots of the subgroup analysis in Group C (PD-1/PD-L1 VS. Placebo): The OR of pneumonitis for all-grade checked using the random effect (RE) model: Subgroup analyses were carried out according to the tumor types and PD-1/PD-L1. (TIF 2504 KB) [file 262_2024_3736_MOESM7_ESM.tif]

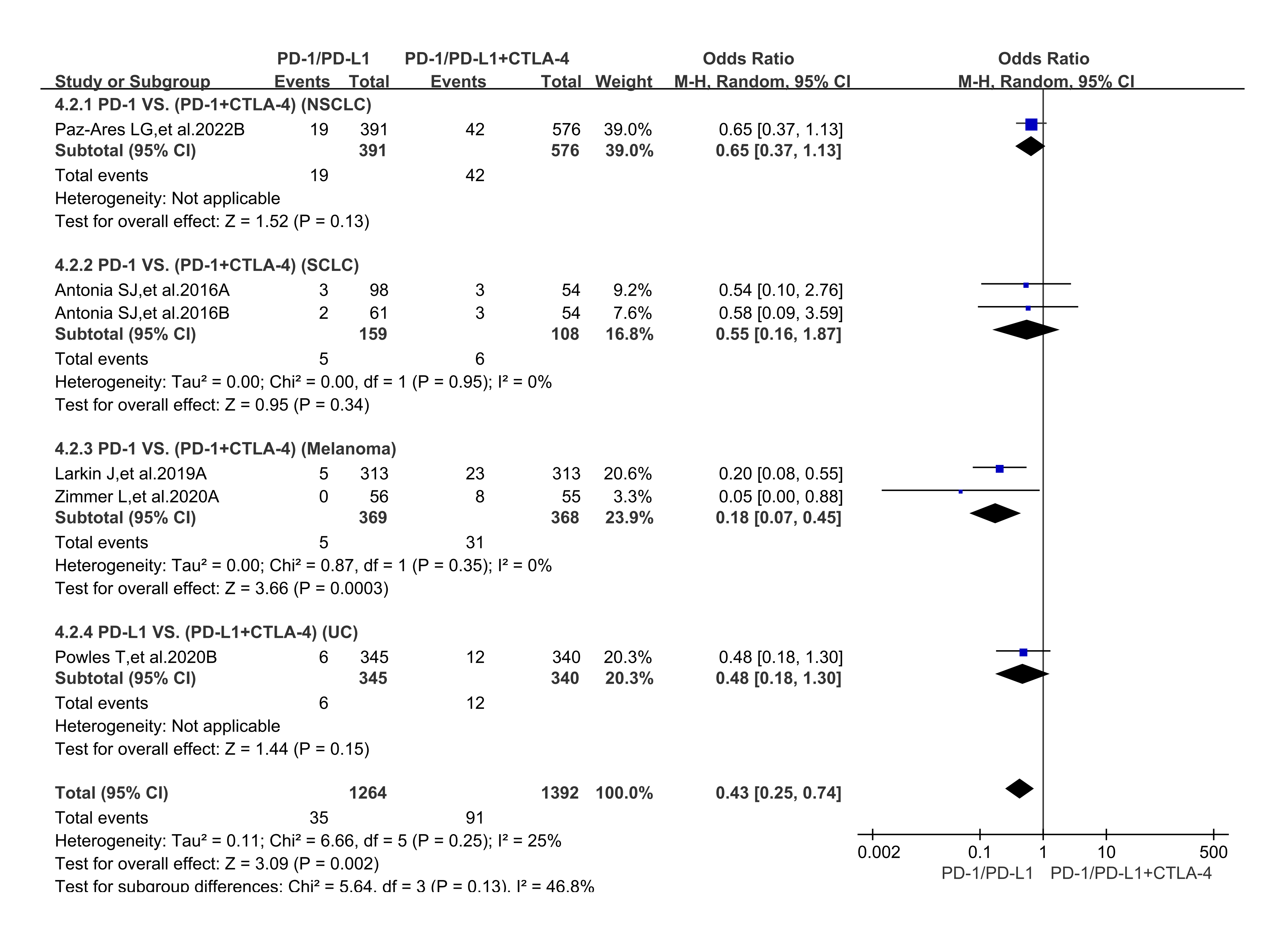

Supplement: Supplementary file 8 — S Figure 7: Forest blots of the subgroup analysis in Group D (PD-1/PD-L1 VS. PD-1/PD-L1+CTLA-4): The OR of pneumonitis for all-grade checked using the random effect (RE) model: Subgroup analyses were carried out according to the tumor types and PD-1/PD-L1. (TIF 1597 KB) [file 262_2024_3736_MOESM8_ESM.tif]
